# Signaling pathways of EBV-induced oncogenesis

**DOI:** 10.1186/s12935-021-01793-3

**Published:** 2021-02-06

**Authors:** Yin Luo, Yitong Liu, Chengkun Wang, Runliang Gan

**Affiliations:** Cancer Research Institute, Medical School, University of South China, Chang Sheng Xi Avenue 28, Hengyang, 421001 Hunan People’s Republic of China

**Keywords:** Epstein‐barr virus (EBV), Cancer, Signaling pathway

## Abstract

Epstein-Barr virus (EBV) is closely associated with multiple human cancers. EBV-associated cancers are mainly lymphomas derived from B cells and T cells (Hodgkin lymphoma, Burkitt lymphoma, NK/T-cell lymphoma, and posttransplant lymphoproliferative disorder (PTLD)) and carcinomas derived from epithelial cells (nasopharyngeal carcinoma and gastric carcinoma). EBV can induce oncogenesis in its host cell by activating various signaling pathways, such as nuclear factor-κB (NF-κB), phosphoinositide-3-kinase/protein kinase B (PI3K/AKT), Janus kinase/signal transducer and transcription activator (JAK/STAT), mitogen-activated protein kinase (MAPK), transforming growth factor-β (TGF-β), and Wnt/β-catenin, which are regulated by EBV-encoded proteins and noncoding RNA. In this review, we focus on the oncogenic roles of EBV that are mediated through the aforementioned signaling pathways.

## Background

As a kind of human pathogen, Epstein-Barr virus (EBV) is closely associated with the development of several cancers. More than 90% of adults have been infected with EBV [1], but as long as equilibrium is maintained between the host and the virus, the infection has no unfavorable effects on the human body. Once human immune surveillance fails, latent EBV can be reactivated to induce abnormal proliferation and transformation of host cells, which can cause EBV-related cancers. The EBV genome encodes three latent membrane proteins (LMP1, LMP2A and LMP2B), five Epstein-Barr nuclear antigens (EBNA1, EBNA2, EBNA3A, EBNA3B and EBNA3C) and one leader protein (EBNA-LP). The EBV genome also encodes two different classes of miRNAs: Bam HI fragment H rightward open reading frame (BHRF1) miRNAs and BamHI-A rightward transcript (BART) miRNAs [2].

LMP1 has been confirmed as an oncoprotein that possesses conversion characteristics in vitro and is regularly expressed in EBV-related cancers. The main regulatory functions of LMP1 are mediated by its C-terminal activating regions (CTAR1, CTAR2 and CTAR3). CD40 and B cell receptors (BCRs) are simulated by LMP1 and LMP2, respectively [3, 4] and are the receptors of tumor necrosis factor (TNF) and key B cell costimulatory receptors [5]. EBNA1 plays an indispensable role in the effective replication of the EBV genome. The combination of EBNA1, nucleolin and nucleophosmin can activate the transcription of EBNA-1 and ensure the persistence of the EBV genome [6]. EBNA-2 and EBNA-LP are critical in converting EBV-infected B cells into lymphoblastoid cell lines (LCLs) and maintaining the resultant LCLs. Furthermore, they synergistically activate the gene transcription of viruses and cellular genes [7, 8]. The EBNA3A, EBNA3B and EBNA3C gene families are highly similar to each other in terms of their promoter and genetic structure and in their way of regulation, as they all play a role in regulating the transcription of host cells [5]. The oncogenic mechanisms of EBV-encoded proteins, especially the oncoprotein LMP1, and the miR-BART family of noncoding RNAs are closely related to the following signaling pathways.

## Six main signaling pathways of EBV-associated neoplasms

### NF-κB signaling pathway

Nuclear factor-κB (NF-κB), a highly conserved family of transcription factors, includes p50, p52, RelA(p65), RelB, and c-Rel [9]. When stimulated, IκB kinase (IKK) phosphorylates serine at the IκB subunit regulatory site of the NF-κB complex so that the IκB subunit can be modified by ubiquitination and then degraded by protease, thereby releasing NF-κB [9]. Free NF-κB enters the cell nucleus to bind the target gene to initialize the transcription process. NF-κB can also activate the expression of IκBα to reinhibit the activity of NF-κB, forming a spontaneous negative feedback loop [10].

**Fig. 1:**
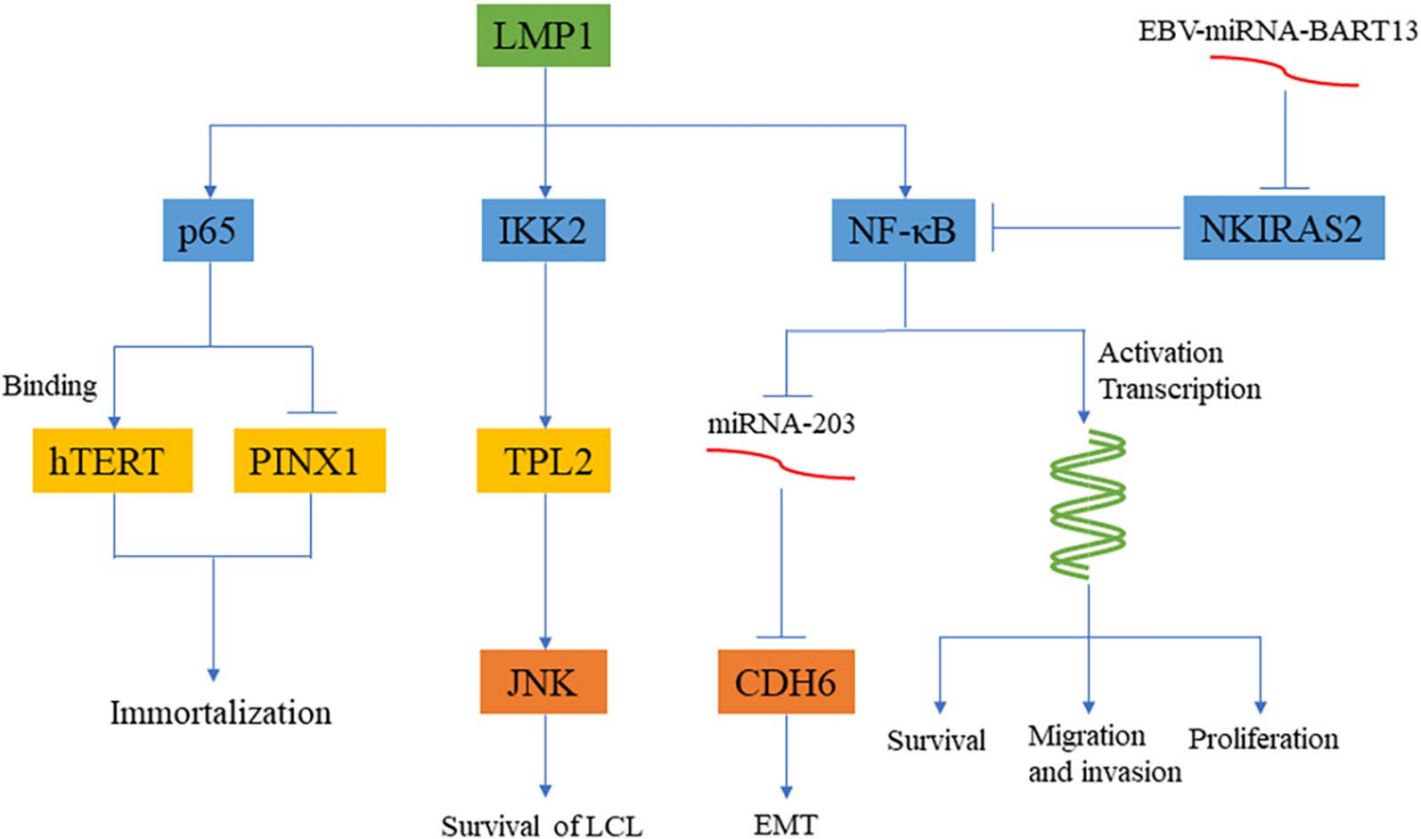
EBV-induced oncogenesis through the NF-κB signaling pathway. LMP1 can enhance the activity of NF-κB, enter the nucleus to activate transcription and promote cell proliferation, migration and invasion. LMP1 can inhibit the expression of miRNA-203 through NF-κB, resulting in high expression of CDH6 and EMT. At the same time, LMP1 can also activate the IKK2/TPL2/JNK signaling axis, which is beneficial to the survival of LCL. The combination of LMP1 and p65 can activate hTERT and inhibit PINX1, which together promote cell immortalization. EBV-miRNA-BART13 inhibits NKIRAS2, thereby promoting cell proliferation, migration and invasion

NF-κB dysregulation is one of the key steps in the occurrence and development of most tumors. The expression of NF-κB is upregulated in some cancers, such as head and neck squamous cell carcinoma (HNSCC) [11], colorectal cancer (CRC) [12], pancreatic ductal adenocarcinoma (PDAC) [13], and gastric cancer (GC) [14]. Among the products of EBV, LMP1 is the most studied. It regulates cell proliferation, apoptosis, transformation, metastasis and invasion through NF-κB (Fig. 1). In nasopharyngeal carcinoma (NPC) cells, LMP1 induces the p65 subunit of NF-κB to bind with human telomerase reverse transcriptase (hTERT) and then activate telomerase. Thus, the final effect of LMP1 inducing NF-κB activation is LMP1-mediated immortalization [15]. Liu et al. [16] discovered that there is an important link between LMP1 and the cancer suppressor gene PIN2 (TERF1)-interacting telomerase inhibitor 1 (PINX1). p65 can bind to the PINX1 promoter to inhibit PINX1 transcription, which leads to the increased proliferation of cancer cells. This finding further shows that LMP1 mediates cell immortalization via NF-κB. Kim et al. [17] found that EBV-mediated B lymphocyte transformation requires assistance from LMP1 and that activation of NF-κB is critical for LCL survival. They also discovered that an ethanol extract of *Chrysanthemum indicum Linné* (CIE) can strikingly reduce LMP1-induced NF-κB activation as well as LCL activity but produces no negative effects on human foreskin fibroblasts (HFFs), EBV-negative Burkitt lymphoma cells or HeLa cells. Further evidence suggests that the LMP1-induced IKK2-TPL2 signaling pathway may mediate JNK signaling to effectively regulate the proliferation and survival of EBV-transformed LCLs [18]. LMP1 can also regulate cell migration and invasion abilities through NF-κB. On the one hand, LMP1 upregulates the expression of NTRK2 through NF-κB to enhance anoikis resistance, while NTRK2 enhances cell epithelial-mesenchymal transition (EMT) through ERK/AKT [19]. On the other hand, a study found that LMP1 is not only transferred to recipient cells through NF-κB activation of the exosomal packaging of LMP1 but also inhibits miR-203 and promotes the expression of CDH6, leading to EMT in NPC cells. Aspirin reversed the NF-κB/miR-203/CDH6/EMT signaling axis in NPC cells [20].

EBV-encoded miR-BARTs also have an important impact on cell proliferation and migration. The total phosphorylation levels of TAK1 and IKKα/β/γ in NPC tissue samples increased correspondingly when EBV-miR-BART8-3p was upregulated. Furthermore, EBV-miR-BART8-3p can regulate NPC cell migration by activating NF-κB and Erk1/2 [21]. Xu et al. [22] detected highly expressed EBV-miR-BART13 in the serum samples of NPC patients, which can directly target NF-κB inhibitor interacting Ras-like 2 (NKIRAS2) to promote the expression of the NF-κB signaling pathway and increase cell proliferation and migration (Fig. 1).

### PI3K/AKT signaling pathway

Phosphoinositide-3-kinase (PI3K), an intracellular phosphatidylinositol kinase, is equipped with serine/threonine activity and phosphatidylinositol kinase activity. When signals are received from tyrosine kinases and G protein-coupled receptors, p85 of the PI3K regulatory subunit is recruited to the site around the cytomembrane. Then, protein kinase B (AKT) translocates from the cytoplasm to the cytomembrane and activates AKT with assistance from PDK1 and PDK2 [23]. The PI3K/AKT signaling pathway is strictly regulated by PTEN in normal cells, but an abnormally activated PI3K/AKT signaling pathway can induce ubiquitination and degradation of PTEN and accelerate the development of tumors [24].

**Fig. 2:**
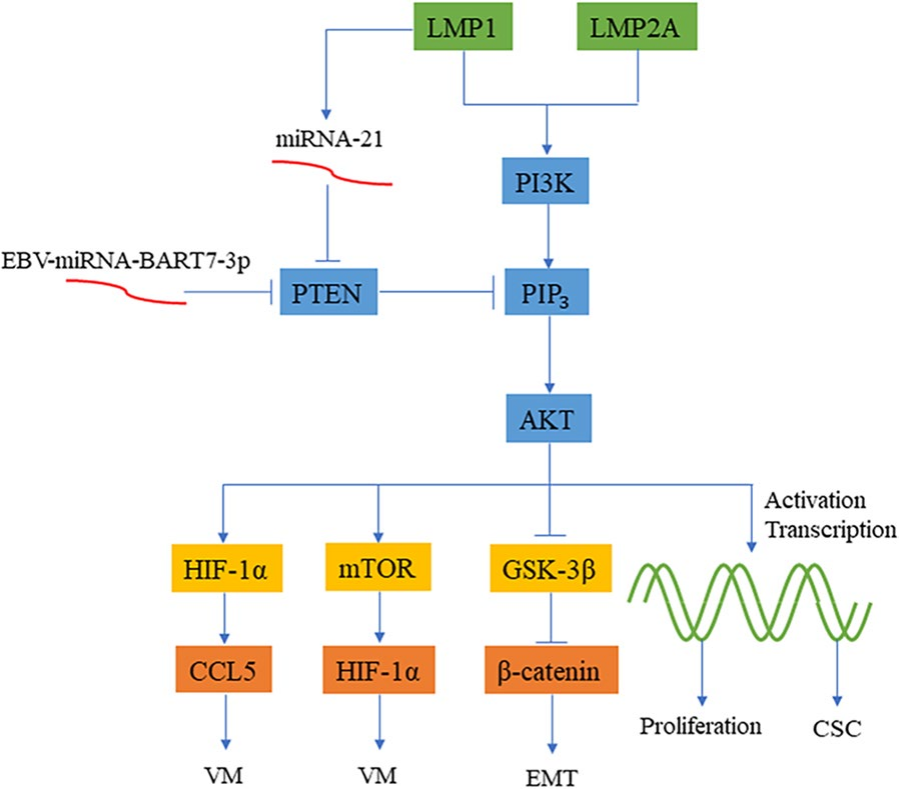
EBV-induced oncogenesis through the PI3K/AKT signaling pathway. LMP1 and LMP2A promote angiogenesis through the PI3K/AKT/HIF-1α/CCL5 signaling axis and the PI3K/AKT/mTOR/HIF-1α signaling axis, respectively. LMP1 inhibits PTEN through miRNA-21 and enhances the PI3K/AKT signaling pathway to promote the formation and proliferation of CSCs. EBV-miRNA-BART7-3P can also promote the high expression of β-catenin by inhibiting PTEN, leading to EMT

The products of EBV can regulate cell proliferation and promote the occurrence of EMT and tumor angiogenesis through PI3K/AKT (Fig. 2). LMP1 is still the most studied hot spot. Wang et al. [25] found that after LMP1 activates Src through subunit p85 of PI3K, src enhances the activity of interferon regulatory factor 4 (IRF4) and promotes the transformation of cells into LCL. IRF4 plays an important role in the differentiation of B cells, T cells and macrophages and is a diagnostic and prognostic marker for different hematological tumors [26]. Meanwhile, LMP1 downregulates the expression level of PTEN by enhancing the expression of miR-21 and then activates PI3K/AKT, thereby stimulating the expression of cancer stem cell (CSC) markers and promoting the development of cancer and the formation of tumor clusters [27]. Angiogenesis is also critical to tumor development, while both LMP1 and LMP2A can induce angiogenesis. LMP1 was found to activate the PI3K/AKT and HIF-1α signaling pathways in EBV-positive NPC cells and to play a key role in chemokine ligand 5 (CCL5)-mediated cancer angiogenesis [28]. CCL5 is considered to be a new therapeutic target. Xiang et al. [29] showed that epithelial carcinoma cells develop a non-VEGF-dependent cancer vascular network after infection with EBV. In their study, LMP2A-mediated activation of PI3K/AKT/mTOR/HIF-1α signaling was an important cause of EBV-induced vasculogenic mimicry (VM) (Fig. 2).

EBV-encoded miRNAs regulate cellular EMT via the PI3K/AKT signaling pathway. Intranuclear accumulation of the oncoprotein β-catenin indicates the initialization of EMT, which can be negatively regulated by GSK-3β. Although GSK-3β and β-catenin are downstream proteins of the WNT signaling pathway, they are often regulated by PI3K/AKT because each signaling pathway does not exist independently. Cai et al. [30] confirmed that EBV-miR-BART7-3p targets the human cancer suppressor gene PTEN in NPC cells, activates the PI3K/Akt/GSK-3β signaling axis and finally induces the high expression and intranuclear translocation of Snail and β-catenin to facilitate EMT (Fig. 2). In another study, EBV-miR-BART22 directly targeted MAP2K4 and upregulated the expression of nonmuscle myosin heavy chain IIA (MYH9) through the PI3K/AKT/c-Jun signaling axis [31]. They demonstrated that the combination of MYH9 with GSK-3β induced the ubiquitin degradation of GSK-3β and promoted the expression and nuclear translocation of β-catenin to induce EMT. A new study analyzed the causes of angiogenesis at the metabolic level, and EBV-miRNA-BART1-5p inhibited AMPKα1 and PTEN in NPC cells, thereby activating the AMPK/mTOR/HIF-1 signaling pathway, enhancing aerobic glycolysis, and promoting abnormal cell proliferation and angiogenesis [32].

However, there is no direct evidence that EBV products regulate the proliferation and apoptosis of posttransplant lymphoproliferative disease (PTLD) through the PI3K/AKT signaling pathway. Many studies have shown that PI3K/AKT signaling pathway plays an important role in PTLD infected by EBV. In the study of Sang et al. [33], several nodes inside the PI3K/AKT/mTOR signaling pathway of EBV-positive PTLD-derived cell lines were activated. Both CAL-101 and MK-2206, specific inhibitors of PI3K/AKT, can extend the survival time of C57BL/6 mice that received a cardiac allograft and, when combined with rapamycin, can inhibit cancer cell growth. The combined therapy of rapamycin and a PI3K/AKT inhibitor can effectively treat EBV-associated PTLD and improve the isograft survival rate. According to Hatton et al. [34], in PTLD-derived EBV-positive B cells, LMP2A activates Syk and prevents the loss of X-linked inhibitor of apoptosis protein (XIAP) through the PI3K/AKT signaling pathway to maintain cell survival, while administration of Syk and PI3K/AKT inhibitors can induce cell apoptosis. Two years later, Hatton et al. [35] indicated that Syk induced a carcinogenic state in an animal model via the PI3K/AKT-dependent signaling pathway. After administration of a PI3K/AKT-specific inhibitor, the animal model showed significantly reduced tumor volume.

### JAK/STAT signaling pathway

In the Janus tyrosine kinase/signal transducer and transcription activator (JAK/STAT) family, there are several members, including JAK1, JAK2 and TYK2, that are commonly expressed in all types of cells. In contrast, JAK3 expression is usually limited to lymphoid cells [36]. The transmission of the JAK/STAT signaling pathway is composed of three parts, namely, the tyrosine kinase-related receptor Janus tyrosine kinase (JAK) and the transcription factor (STAT). After activation by JAK phosphorylation, the STAT dimer exposes the nuclear localization signal (NLS) and enters the nucleus to take part in gene expression. In normal cells, the activation of the JAK/STAT and NF-κB signaling pathways is strictly controlled. The activation of the JAK/STAT and NF-κB signaling pathways is considered to be a characteristic of EBV-positive diffuse large B-cell lymphoma (DLBCL) patients, while the expression of JAK/STAT and NF-κB signaling is not obvious in EBV-negative DLBCL patients [37]. Additionally, in EBV-positive tumors, promoter report analysis has fully proven that the two promoters of LMP1, L1-TR and ED-L1, both directly bind with STAT after activation upstream of JAK/STAT to change the normal function of cells [38].

**Fig. 3:**
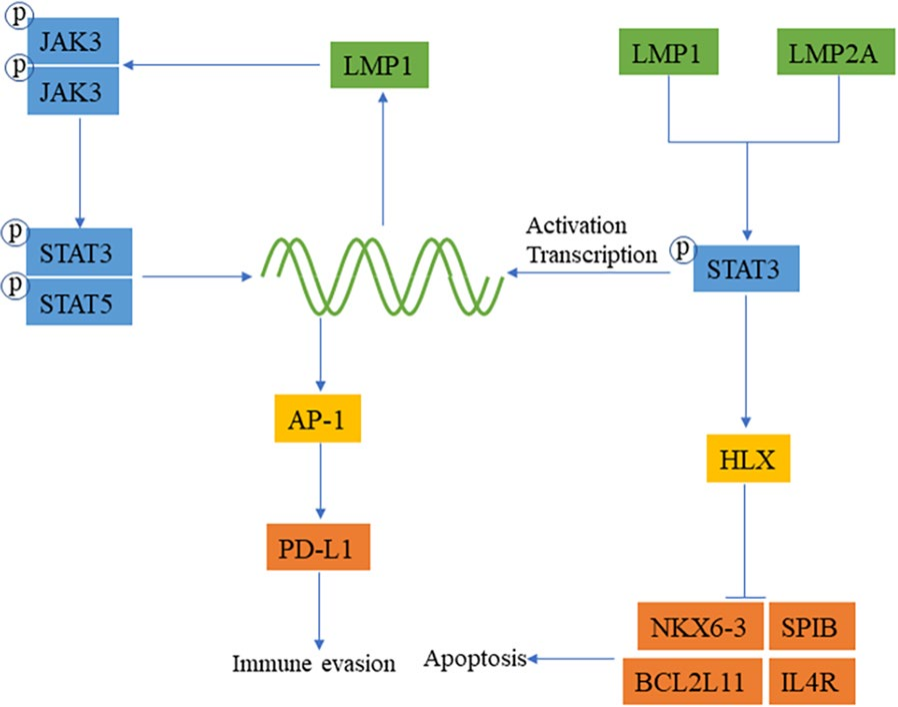
EBV-induced oncogenesis through the JAK/STAT signaling pathway. When JAK is activated, it can phosphorylate STAT, and then dimeric STAT is exposed to the nuclear signal and enters the nucleus to participate in regulating gene expression. LMP1 can activate JAKs and then activate the STAT dimer, which promotes the corresponding transcriptional regulation in the nucleus and can provide feedback and enhance the regulation of its own expression. LMP1 enhances the transcription of AP-1 through JAK/STAT and promotes the expression of PD-L1 to avoid immune surveillance. LMP1 and LMP2A can activate HLX to inhibit apoptosis

To date, 7 independent STAT proteins have been identified that are closely related to apoptosis and immune evasion. On the one hand, evidence suggests that STAT3 and STAT5 control the cell cycle proceeding from G1 to S phase by raising the expression level of cyclin D1 [39, 40]. Other researchers have also observed that STAT3 is activated by LMP1 and LMP2A in DLBCL and upregulates the expression of HLX, which inhibits NKX6-3, SPIB, IL4R and BCL2 L11 to interfere with cell differentiation and inhibit cell apoptosis [41] (Fig. 3). On the other hand, JAK/STAT signaling usually mediates the expression of PD-L1 to help cancer cells escape surveillance of the human immune system. Amplification of chromosome 9p24.1 enhances JAK2 expression to induce the JAK2/STAT1 signaling pathway and activates PD-L1 expression to evade attack from the immune system [42]. Based on previous studies, LMP1 activates JAK3 and related STAT proteins to improve the activity of the AP-1-dependent PD-L1 enhancer and to upregulate PD-L1 in EBV-positive classical Hodgkin lymphoma (cHL) cells [43]. These studies imply that JAK/STAT-mediated PD-L1 expression evades surveillance of the immune system.

### MAPK signaling pathway

Mitogen-activated protein kinases (MAPKs) are involved in helping direct cellular responses to various extracellular stimuli, such as cytokines, neurotransmitters, and hormones, as well as cellular stress and adhesion. In the MAPK signaling pathway, there is a conservative pattern of three kinases, including MAPK kinase kinase (or MAP kinase kinase kinase, MKKK), MAPK kinase (or MAP kinase kinase, MKK) and MAPK, which are activated in turn when stimulated. There are 4 major classes of MAPKs, namely, ERK, p38, JNK and ERK5. Among those classes, both ERK1 (MAPK3) and ERK2 (MAPK1) are involved in growth factor signal transmission and cell proliferation and apoptosis [44].

**Fig. 4:**
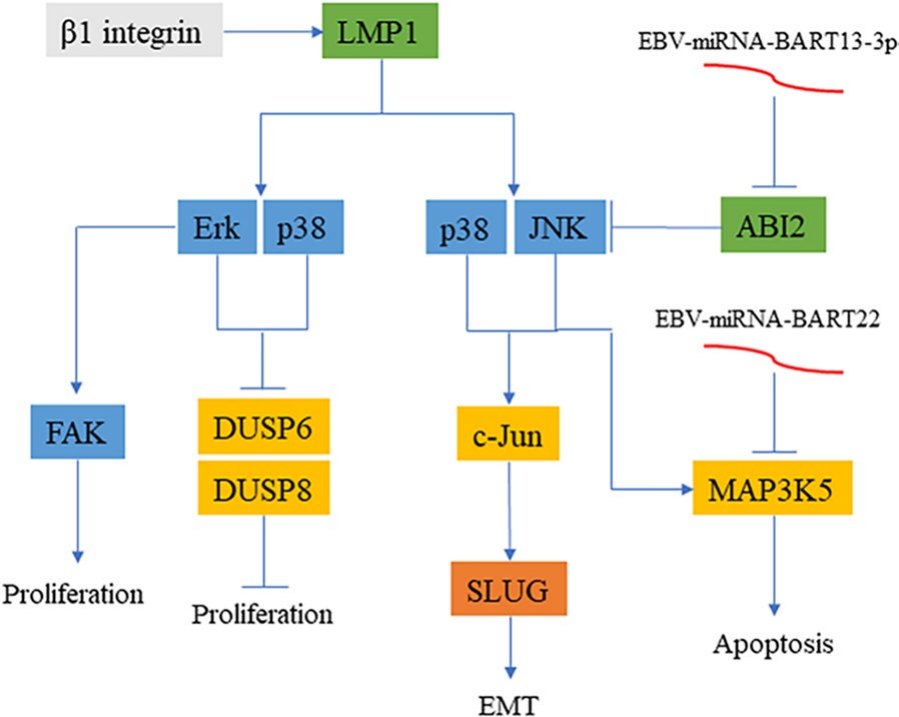
EBV-induced oncogenesis through the MAPK signaling pathway. LMP1 can activate ERK, JNK, and p38 and reduce the expression of DUSP6 and DUSP8 to promote cell proliferation and enhance c-JUN to promote the occurrence of EMT. β1 integrin can promote the expression of LMP1 and activate FAK, which will promote cell proliferation. EBV-miRNA-BART13-3P and EBV-miRNA-BART22 inhibit the upstream and downstream MAPK pathways, promote EMT, and resist apoptosis, respectively

EBV-encoded products share some close associations with the MAPK signaling pathway (Fig. 4). LMP1 not only inhibits the expression of dual-specific phosphatase 6 (DUSP6) and DUSP8 through ERK and p38 in the MAPK signaling pathway and promotes the proliferation of LCL but also affects other cells through extracellular vesicles (EVs) and activates the p38-MAPK signaling pathway in NPC cells, enhancing the resistance of cells to chemotherapy [45, 46]. Analysis of the expression of virus-encoded proteins showed that the use of p38-MAPK inhibitors reduces the expression of BZLF1 and BGLF2-induced virus replication, indicating that EBV can enhance its own infection ability through the p38-MAPK signaling pathway [47]. Moreover, EBV-encoded products may regulate cellular EMT by means of the MAPK signaling pathway. Morris et al. [48] showed that β1 integrin could strengthen LMP1-mediated ERK-MAPK and focal adhesion plaque (FAK) phosphorylation to improve cell viability. They also demonstrated in an MDCK canine model that LMP1 could promote EMT. The BART family also promotes EMT and migration invasion, EBV-miR-BART13-3p could inhibit the expression of ABI2 and activate the c-Jun/SLUG signaling pathway through the p38-MAPK/JNK signaling pathway so that EMT could increase NPC transfer [49]. EBV-miRNA-BART22 promotes NPC cell migration and invasion and inhibits cell apoptosis by targeting ASK1 (MAP3K5), a downstream component of the p38-MAPK signaling pathway [50].

The JNK/p38-MAPK signaling pathway plays a vital role in cell apoptosis, especially oxidative stress-induced cell apoptosis. In a study on reactive oxygen species (ROS), high expression of CD70 on EBV-transformed B cells could also evoke the stress reaction of the endoplasmic reticulum (ER), and the JNK and p38-MAPK signaling pathways may interact to induce ROS generation and subsequent cell apoptosis [51]. Further research showed that sorafenib can stimulate EBV-transformed cells to form ROS to activate JNK/p38-MAPK, which can translocate Bcl-2-associated X protein (BAX) into the mitochondria to activate caspase-9 and induce apoptosis with caspase-3 [52]. The aforementioned phenomena were observed in EBV-transformed cell lines, and the specific role of EBV products has been gradually discovered. ROS activation is regulated in different ways through latent EBV infection. For instance, in type III latency, EBNA2 can activate the transcription of LMP1 to induce ROS production [53]. However, more experimental evidence is needed to understand how EBV-encoded products participate in the regulation of ROS production and control cell apoptosis.

### TGF-β signaling pathway

The transforming growth factor-β (TGF-β) signaling pathway is involved in many cellular processes of both mature organisms and developing embryos. Such processes include cell growth, differentiation, apoptosis and dynamic equilibrium. TGF-β superfamily ligands may bind with the TGF-βII receptor, while the II type receptor is a kind of serine/threonine kinase receptor that catalyzes the phosphorylation of the I type receptor that regulates Smad protein expression in return. On the other hand, Smad proteins may change the phenotype via regulation and transcription [54].

**Fig. 5:**
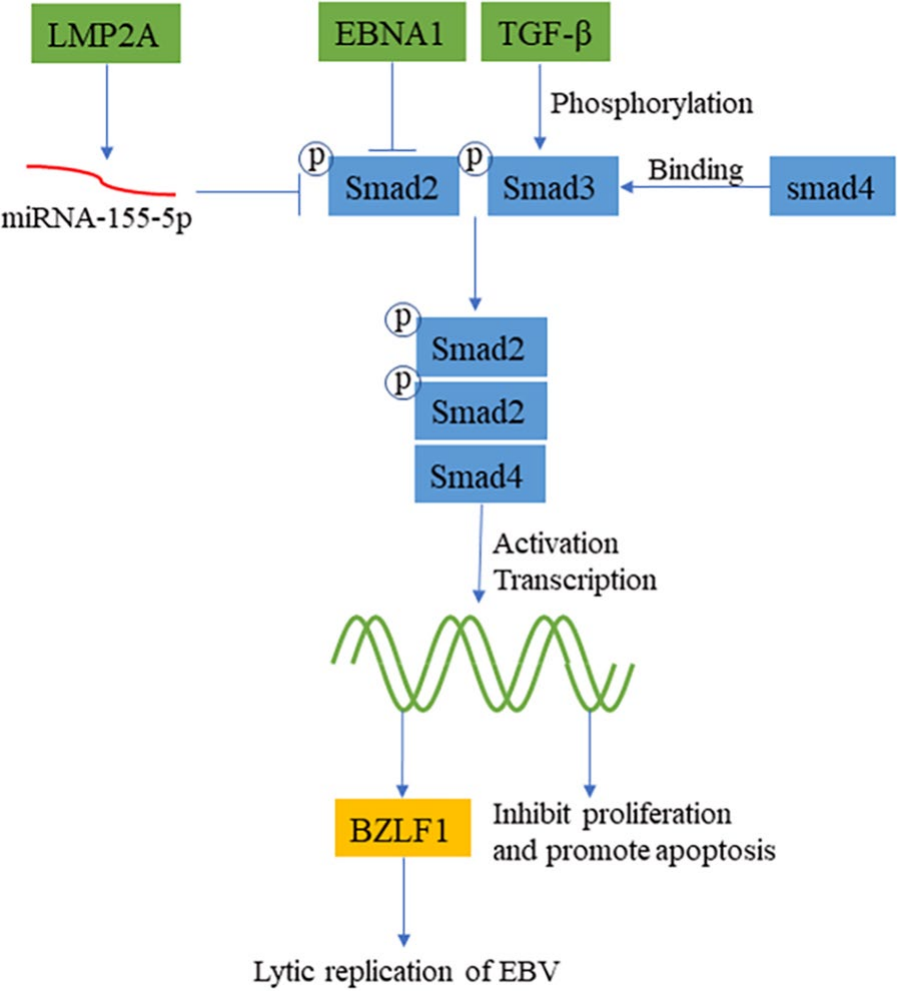
EBV-induced oncogenesis through the TGF-β signaling pathway. After TGF-β is stimulated, it activates Smad2 and Smad3 and then combines with Smad4 to enter the nucleus to regulate transcription, inhibit cell proliferation signals, and promote apoptosis of injured cells. LMP2A and EBNA1 can destabilize Smad2, thereby inhibiting the proliferation-inhibiting signal produced by TGF-β. Cells that have been infected with EBV can promote the activation of the TGF-β signaling pathway and increase the expression of the BZLF1 gene, leading to increased viral replication and enhanced infectivity of EBV

In studying the regulation of TGF-β on apoptosis and proliferation, EBV-positive NPC cells can resist TGF-β1-mediated growth inhibition and apoptosis, while the growth of EBV-negative NPC cell lines is strictly regulated by TGF-β1 [55], among which EBNA1 and LMP2A may play important roles (Fig. 5). Evidence has revealed that the expression of EBNA1 in the nasopharyngeal adenocarcinoma cell line Ad/AH could inhibit TGF-β1 transcription by reducing the binding of Smad2 to Smad4 [56]. At the same time, EBNA1 can promote the degradation of Smad2 protein and inhibit the transcription of TGF-β target gene protein tyrosine phosphatase receptor κ (PTPRK), thereby promoting the growth and survival of Hodgkin lymphoma (HL) cells [57]. Shi et al. [58] found that LMP2A activates miR-155-5p and inhibits the phosphorylated Smad2 protein through NF-κB to eliminate the effect of TGF-β on growth inhibition. LMP1 upregulated the activity of calprotectin (CRT) in NPC cells to induce EMT. When CRT is knocked out, the LMP1-mediated TGF-β/Smad3/NRP1 signaling pathway is impaired, and NPC metastasis and invasion are reduced accordingly [59].

EBV could also strengthen its own infection and transmission through the TGF-β signaling pathway. TGF-β activates Zp through the combination of AP-1 and Smad4. Activated Zp promotes the expression of BZLF1, causing EBV to switch from a latent form of infection to a lytic form of infection [60]. According to Iempridee et al. [61], TGF-β-induced reactivation of EBV involves the expression of Smad2, Smad3 and Smad4 as well as joint lytic replication-related BZLF1 gene expression. The above is one aspect, from another perspective. Nanbo et al. [62] discovered in their study that epithelial cells infected with EBV could spontaneously secrete TGF-β, and the expression and sensitivity of EBV infection in epithelial cells is enhanced by cell-to-cell contact and thus induces the replication of intracellular viruses. The above findings partly explain why TGF-β promotes the transmission of EBV.

### Wnt/β-catenin signaling pathway

The classical Wnt signaling pathway can regulate genetic transcription. As a kind of Wnt signaling pathway, Wnt/β-catenin can signal β-catenin to gather within the cytoplasm, translocate to the nucleus and bind with TCF/LEF family transcription regulatory factors to exert a corresponding regulatory effect. Without Wnt, β-catenin cannot gather inside the cell due to ubiquitylation and degradation by protein complexes (Axin, APC, Dvl, GSK3 and CK1). These protein complexes may encourage ubiquitylation of β-catenin first and then transport it to the proteasome for digestion. In contrast, the presence of Wnt promotes the dissociation of the protein complex and loses the ability to ubiquitinate β-catenin, resulting in stable expression of β-catenin [63].

**Fig. 6:**
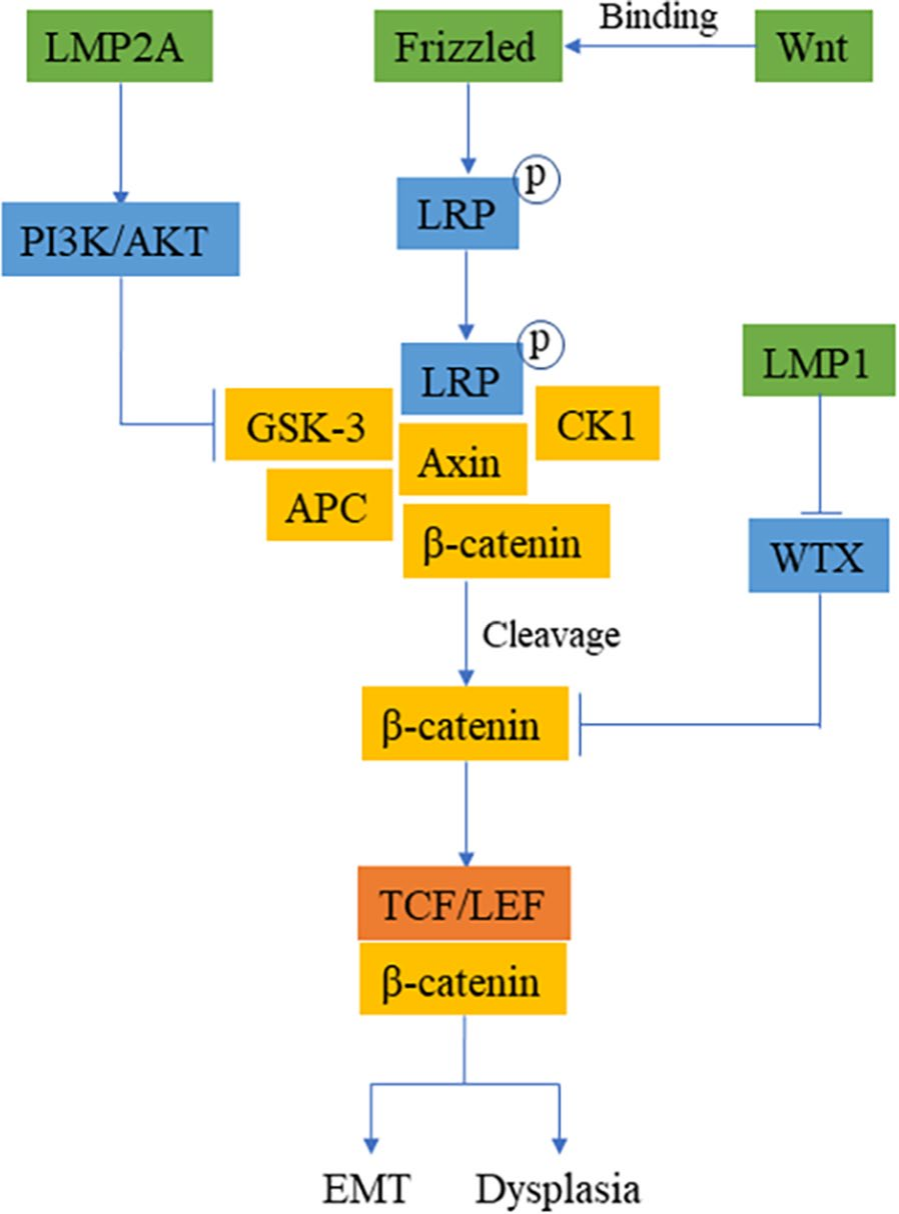
EBV-induced oncogenesis through the Wnt/β-catenin signaling pathway. The binding of Wnt and Frizzled protein will prompt LRP to phosphorylate and recruit protein complexes, and then these protein complexes will dissociate to release stable expression of β-catenin. Free β-catenin binds to TCF to activate the corresponding transcription after entering the nucleus. LMP2A inhibits the expression of GSK-3β through the PI3K/AKT signaling pathway, which interferes with the formation of protein complexes and promotes the accumulation of β-catenin in the nucleus to induce EMT. WTX can inhibit the expression of β-catenin, and LMP1 in transgenic mice will interfere with the inhibitory effect of WTX, resulting in the stable expression of β-catenin and leading to dysplasia

According to previous studies, LMP1 is unlikely to play a vital role in Wnt signaling pathway regulation [64], but some studies have proven the ability of EBV-associated proteins to function in regulating the biological functions of cells through the Wnt signaling pathway (Fig. 6). Qing et al. [65] demonstrated that LMP1 could inhibit WTX and enhance the steady expression of β-catenin in HNE-1 cells. Moreover, LMP1 transgenic mice showed significant epithelial cell dysplasia in the nasopharynx, oropharynx, stomach, and kidney. Although no tumors were induced, preliminary evidence indicated that LMP1 could regulate cell proliferation via the Wnt signaling pathway. The Wnt signaling pathway is closely related to EMT, and only a few studies have shown that EBV products are involved. The Wnt signaling pathway can induce EMT by activating phosphorylation mediated by the combination of LRP and GSK-3β and by inhibiting the degradation of β-catenin in the cytoplasm [63]. Experiments have shown that LMP2A activates PI3K/AKT to inhibit the downstream GSK-3β of Wnt and stops protein complexes from forming, enabling the stable accumulation of β-catenin in the nucleus, which induces EMT [66, 67]. However, more experiments are needed to prove the connection between EBV-encoded products and the Wnt signaling pathway.

With the advancement of medicine and application science, the synergistic effect and interaction at the molecular level are frequently unveiled and confirmed. EBV-encoded products may activate various signaling pathways, such as the NF-κB, JNK and JAK/STAT signaling pathways. These signaling pathways collectively regulate the interactions among specific downstream proteins, providing evidence for the oncogenic mechanism of EBV-encoded products. The synergistic effect between NF-κB and PI3K/AKT in LMP1-promoted aerobic glycolysis, as they interact with each other to enhance the transcription and plasma membrane translocation of glucose transporter 1 (GLUT1) and thus fuel the proliferation of cancer cells [68]. In addition, through the JAK/STAT and MAPK/ERK signaling pathways, LMP1 induces the expression of vascular endothelial growth factor (VEGF) and promotes angiogenesis [69]. In a study of the ability of EBV to regulate metastasis, LMP1 enhanced the expression of Capn4 through JNK/AP-1 and cooperated with LMP1 to enhance the activity of NF-κB and promote actin rearrangement to enhance NPC metastasis [70]. Moreover, LMP2A inhibits TGF-β1-induced apoptosis by means of the PI3K/AKT signaling pathway [71]. In another study, the EBV genome was transfected into HONE1 cells. The JAK/STAT signaling pathway was significantly inhibited, whereas the NF-κB and PI3K/AKT signaling pathways were enhanced in HONE1 cells. The direct effect on cells can be illustrated by enhanced cell proliferation and migration, accelerated cell cycle progression, and inhibited cell apoptosis [72]. A comprehensive examination of various signaling pathways could be helpful for making correct decisions regarding treatment.

## EBV products affect other cells through EV

LMP1 could be active in all the signaling pathways mentioned above. From a large number of experiments on LMP1, LMP1 promotes the generation of extracellular vesicles (EVs), which has attracted increasing attention. Therefore, more experiments are needed to understand these mechanisms at the molecular level. LMP1 upregulates the expression of CD63, Alix, Syntenin-1, Hrs, TSG101 and CHMP5 through the endosomal sorting complex required for transport (ESCRT), increasing the production of EV [73]. CD63 is the key molecule in this process, as CD63 regulates the packaging of LMP1 exosomes and interacts with CTAR1 through ubiquitin C-terminal hydrolase L1 (UCH-L1) to introduce LMP1 into EVs. The combination of EVs and other receptor cells changes the normal NF-κB signal [74, 75]. Another study showed that by regulating the NF-κB signaling pathway, LMP1 upregulates the expression of syndecan-2 (SDC2) and synaptotagmin-like-4 (SYTL4) in NPC cells to promote the secretion of LMP1-containing EVs. This process not only enhances the growth and proliferation of cancer cells in vivo but is also closely related to the migration and invasion of cancer cells [76]. As shown by the study of Wu et al. [77], CNE1 cells may secrete LMP1-containing EVs, which affect the NF-κB signaling pathway of other cells so that normal fibroblasts (NFs) can be transformed into cancer-associated fibroblasts (CAFs), and the metabolic activity as well as migration and invasion of these cells are enhanced. In addition, there are a number of viruses and their miRNAs in EVs secreted by EBV-infected B cells [78], which can affect the normal biological activities of uninfected cells, similar to LMP1. Similar to EBERs, LMP2A can also exert corresponding effects via exosomes [78]. Whether other EBV-encoded products have similar functions needs to be further investigated. Importantly, a deeper understanding of the relationship between EBV-encoded products and EVs may provide a new way to develop new therapeutic strategies. The noncoding RNA produced by EBV has been increasingly confirmed to be involved in suppressing host immune-related genes and helping EBV-infected cells escape the surveillance of the host immune system [79–81]. The development of drugs targeting noncoding RNAs to prevent immune escape and malignant proliferation of host cells is an ideal treatment option.

## Therapeutic strategies for EBV-induced cancer

After elucidating the mechanism of so many EBV activation signaling pathways, researchers designed inhibitors of certain key proteins in the activation signaling pathway, which has become a method of treating tumors. In the new clinical treatment strategy, the focus is no longer on drug therapy, and cellular immunotherapy has become a hot spot (Table 1). First, targeted stimulation of CTLs in vitro through LMP1/LMP2 and reinfusion to treat patients can stimulate the expansion of LMP1/LMP2 specific T cells [82–84]. In addition, through artificial modification to construct EBNA1/LMP1/LMP2A-sensitive T cells, reinfusion therapy patients effectively recognize the products of EBV and increase the proportion of EBV-specific T cells in the patient [85, 86]. Finally, using recombinant vaccinia virus, MVA-EL as a vaccine, stimulating patients to produce specific antibodies against EBNA1/LMP2 has a certain effect [87]. In addition, by chemically synthesizing DNAzymes targeting LMP1, it can effectively degrade the mRNA of LMP1 in cells and prevent the LMP1 oncoprotein from functioning [88]. However, traditional drug treatments for abnormal proteins in cancer are still important (Table 1). In the EBVaGC cell line, the inhibition of JAK2, PI3K and mTOR by fedratinib, AZD1480 and LY294002, respectively, can inhibit the expression of PD-L1, thereby preventing cancer cells from evading surveillance of the immune system [89]. In one study, LMP1 facilitated NF-κB activation in EBV-positive T and NK cells, and cells processed with the NF-κB-specific inhibitor IMD-0354 had lower proliferation rates and increased apoptosis [90]. In another study, the HIV protease inhibitor ritonavir inhibited the expression of NF-κB in EBV-LCLs and promoted the apoptosis of LCLs. Unfortunately, the relationship between NF-κB inhibition and apoptosis was not further explained. HSPs play an important role in cell proliferation, differentiation and canceration [91]. In principle, drugs developed for HSPs interact with HSPs and destroy their association with the chaperone and substrate proteins of HSPs. AT13387 is a specific inhibitor of Hsp90 that can inhibit the expression of AKT/p-AKT, EGFR and p-STAT3 in EBV-positive nasopharyngeal carcinoma cells, thereby restoring p27 to inhibit cancer cell proliferation [92]. 2-Phenylethynesulfonamide (PES) is a specific inhibitor of HSP70. It can inhibit the expression of EBNA1 and weaken the proliferation of the virus. It can also reduce the expression of AKT and promote the expression of caspase-3, thereby inhibiting the proliferation of cancer cells and promoting apoptosis [93]. The inhibitors mentioned in these studies were developed based on specific proteins, but even patients with the same cancer type may have different molecular types, so the inhibitors used cannot be generalized. In the future, they must be targeted for individual treatment. From the perspective of patient-specific genomes and different molecular subtypes of patients, these data pave the way for precision treatment.

**Table 1:** Clinical and experimental therapeutic strategies for EBV-induced cancer

Therapy/drug	Target	Function	Cancer/disease	References
CTL	LMP1/LMP2	Stimulates LMP-specific T cell expansion	NK/T cell lymphoma	[82–84]
TCR	EBNA1/LMP1/ LMP2A	Stimulate and expand EBV-specific CD8 + T cells in patients	Progressive multiple sclerosis	[85]
Recombinant vaccinia virus	ENBA1/LMP2	Promote the production of EBNA1/LMP2 antibodies	Nasopharyngeal carcinoma	[87]
DNAzyme	LMP1	Targeted degradation of LMP1 mRNA	Nasopharyngeal carcinoma	[88]
Fedratinib, AZD1480 and LY294002	JAK2, PI3K and mTOR	Inhibit the expression of PD-L1 and restore immune surveillance	Gastric cancer	[89]
IMD-0354	NF-κB	Inhibit proliferation and increase apoptosis	NK/T cell lymphoma	[90]
AT13387	Hsp90	Inhibit the expression of AKT/p-AKT, restore the function of p27 and inhibit cancer cell proliferation	Nasopharyngeal carcinoma	[92]

There is an urgent need for new strategies for the treatment of EBV-induced cancer in the clinic. Traditional drug treatments often only target cancer but ignore that even the same cancer-induced factors can be completely different. We urgently need to understand the molecular mechanism, develop drugs for abnormal proteins or genes, and pave the way for individualized precision treatment. Moreover, it is a promising strategy to design specific artificially modified T cells to recognize EBV oncoproteins combined with clinical drugs to alleviate or even cure EBV-induced cancer. Unfortunately, the current technology, artificially modified T cells, will produce serious toxicity and side effects in the human body, which is another problem that urgently needs to be solved. However, we believe that in the near future, after we deeply understand the mechanism of toxic and side effects, we can minimize the occurrence of toxicity and side effects, so that artificially modified immune cells become a powerful tool for us to fight diseases. At the same time, combined with our research on the signaling pathways of disease onset, a real era of molecular individual therapy will come. This is also our outlook on the positive attitude of the future.

## Conclusions

EBV-encoded products greatly affect the cell cycle, proliferation and apoptosis via six major signaling pathways, which exhibit a considerable amount of cross-talk with each other and thereby form a network. Discoveries of these signaling pathways may provide new directions and targets for the prevention and treatment of EBV-associated cancers. Nevertheless, there are certain limitations of studies on the relationships between EBV and JAK/STAT and Wnt/β-catenin, and more evidence is needed to prove the interaction of EBV with MAPK. In this review, we disclosed the regulatory effect of EBV in host cells and cell interactions via multiple signaling pathway networks. It is meaningful for us to further explore the relations between EBV and these signaling pathways to fully understand EBV-induced oncogenesis.

## Data Availability

Not applicable.
